# Beyond “It’s Just a Phase”: A Review of Picky Eating in Children

**DOI:** 10.3390/nu18081247

**Published:** 2026-04-15

**Authors:** Pedro Alarcon, Yvan Vandenplas

**Affiliations:** 1 Department of Pediatric Gastroenterology & Nutrition, British American Hospital, Lima 1090, Peru; alarcon.pedro@hotmail.com; 2 KidZ Health Castle, UZ Brussel, VUB, Laarbeeklaan 101, 1090 Brussels, Belgium

**Keywords:** picky eating, food selectivity, clinical phenotypes, functional impact, dietary progression, implementation gap

## Abstract

Picky eating is one of the most frequent feeding problems in childhood and is often dismissed as a normal developmental phase. Despite a steadily expanding body of research, uncertainty persists regarding its clinical relevance, assessment, and management. This review synthesizes recent evidence on picky eating in children, with a specific focus on definitions, epidemiology, developmental trajectories, underlying mechanisms, clinical impact, and interventions. Reliance on broad definitions and prevalence estimates has obscured clinically meaningful distinctions between transient, developmentally typical food selectivity and persistent patterns associated with nutritional risk, functional impairment, and family stress. Drawing on contemporary data, we propose a continuum-based, phenotype-oriented framework that emphasizes persistence, severity, and functional impact rather than food refusal alone. Advances in understanding picky eating have not consistently translated into improved clinical care, highlighting persistent gaps in implementation, access, and dissemination of evidence-based feeding guidance. Finally, we outline priorities for future research and practice aimed at improving outcomes for children with clinically relevant picky eating.

## 1. Introduction

Picky eating remains one of the most challenging topics in pediatric nutrition and feeding behavior [1]. Although widely recognized by parents and clinicians, picky eating lacks a universally accepted definition and clear diagnostic boundaries [1,2]. In clinical practice, picky eating is often framed as a benign and developmentally normal phase. In contrast, research has variably described it as a behavioral trait, a risk marker for later eating difficulties, or an early manifestation of more complex feeding disorders [1,2,3]. This lack of conceptual alignment helps explain why the topic continues to generate debate and inconsistent clinical responses.

High reported prevalence has further reinforced the perception that picky eating is of limited clinical importance. Population studies consistently report prevalence estimates between 20% and 50% in early childhood [1,4]. These figures are frequently interpreted as evidence that picky eating is largely harmless and self-limited. Prevalence alone, however, does not capture severity, persistence, or functional impact [1,5]. Longitudinal data show that while many children display picky eating behaviors at a single time point, only a minority develop persistent difficulties associated with nutritional risk, family stress, or impaired social functioning [6,7]. The inability to differentiate between these trajectories has reduced the clinical usefulness of much of the existing literature.

In this context, it is important to distinguish between eating behaviors—which refer to child-driven patterns such as picky eating or food fussiness—and feeding behaviors, which describe caregiver practices such as pressure, restriction, or responsive feeding. Picky eating can be conceptualized as a child’s eating behavior with biological and temperamental predispositions that interact dynamically with the feeding environment [1,2,8].

The aim of this state-of-the-art review is to examine how the understanding of picky eating in children has evolved and to assess whether this growing body of evidence has translated into better care for children and families. Specifically, we review current definitions and conceptual frameworks [1,2], summarize evidence on epidemiology, developmental trends, mechanisms, and clinical impact [1,4,5,6,7,8,9], and explore why progress in research has not consistently resulted in improved clinical outcomes [3,10].

In doing so, this review aims not only to synthesize existing evidence, but also to propose a clinically grounded framework linking phenotypic heterogeneity, functional impact, and implementation into everyday pediatric practice.

## 2. Search Strategy

Although this is a narrative, state-of-the-art review, a structured literature search was performed to inform the synthesis. Relevant articles were identified through PubMed, Scopus, and Web of Science using combinations of the terms “picky eating,” “food selectivity,” “feeding behavior,” “child nutrition,” and “feeding disorders.” Priority was given to studies published in the last decade, particularly longitudinal studies, clinical trials, and recent reviews. Additional references were identified through citation tracking. Articles were selected based on relevance to definitions, epidemiology, mechanisms, clinical impact, and management.

## 3. Definitions and Conceptual Framework

### 3.1. Historical Definitions

The term picky eating has been used for decades to describe feeding behaviors characterized by food refusal, limited food variety, or reluctance to try new foods [1]. Early descriptions were largely descriptive and focused on observable behavior, often reflecting parental concern rather than standardized criteria [1,2]. Over time, picky eating has been used interchangeably with terms such as fussy eating, selective eating, and food neophobia, despite important conceptual differences among these constructs [1].

Most historical definitions did not explicitly address duration, severity, or functional/practical consequences/implications [1,2]. As a result, picky eating has frequently been implemented using single parental questions or brief questionnaire items, with boundaries that vary widely across studies and cultures [1,4]. Even in more recent research, definitions continue to rely primarily on parental perception rather than objective measures of dietary adequacy, functional impairment, or need for intervention [4,7,11].

### 3.2. Conceptual Distinctions

Although often used interchangeably, key concepts differ in scope and implication. Food neophobia refers specifically to the reluctance to try unfamiliar foods, typically emerging as part of normal development. Picky eating is broader and includes rejection of both familiar and unfamiliar foods, often resulting in limited dietary variety. Selective eating represents a more persistent, severe, and restrictive form of food avoidance compared to typical, transitory “picky eating,” often characterized by a very narrow range of acceptable foods and a rigid adherence to specific textures, tastes, or brands [12].

These distinctions are not merely semantic but have practical clinical implications. For example, food neophobia typically reflects a developmentally expected response to unfamiliar foods and may respond well to repeated exposure over time. In contrast, broader picky eating involves rejection of both familiar and unfamiliar foods and may require more structured behavioral strategies. More restrictive patterns, often described as selective eating, may signal underlying sensory sensitivity or behavioral rigidity and warrant closer monitoring for persistence and functional impact [13].

Recognizing these differences allows clinicians to better interpret parental concerns, avoid overgeneralization, and tailor guidance according to the underlying pattern of eating behavior rather than applying a uniform approach to all children described as “picky eaters” [1,4,6,8,14].

### 3.3. Why Lack of Consensus Matters

The absence of a shared definition has had important implications for both research and clinical practice. From a scientific standpoint, the absence of a shared definition has (i) limited comparability across studies, (ii) contributed to wide variation in prevalence estimates, and (iii) overlooked the identification of clinically significant subgroups [1,4]. Broad definitions tend to capture large numbers of children with transient, developmentally typical food selectivity, thereby diluting associations with nutritional or psychosocial outcomes [1,6]. This has contributed to apparently conflicting conclusions regarding the significance of picky eating [1,7].

Clinically, a lack of consensus has encouraged antagonizing approaches. Picky eating is often dismissed as a normal phase requiring reassurance alone, while in other cases it is prematurely medicalized with unnecessary investigations or nutritional supplementation [3,10]. Inconsistent terminology also obscures the boundary between picky eating and recognized feeding disorders, delaying appropriate referral for children whose difficulties are persistent or have functional impairment [10,15].

### 3.4. A Proposed Clinical Continuum Model

To address these limitations, picky eating can be conceptualized as a spectrum [1,6]. At one end lies developmentally typical picky eating, characterized by age-appropriate selectivity, normal growth, and minimal functional impact. At the other extreme are feeding disorders associated with nutritional compromise and significant psychosocial impairment.

Within this spectrum, it is also important to recognize potential overlap with clinically defined feeding disorders such as avoidant/restrictive food intake disorder (ARFID) and Pediatric Feeding Disorder (PFD), which are characterized by persistent restrictive intake and clinically significant consequences [10].

This sequence should not be interpreted as a rigid progression, but rather as a conceptual framework reflecting increasing severity, persistence, and functional impact.

Actually, movement along this continuum is dynamic and influenced by the interaction between child-specific factors (such as temperament, sensory sensitivity, and biological predisposition), caregiver feeding practices, and broader environmental context. Children may move in either direction over time, with improvement occurring in supportive, low-pressure environments and persistence or worsening more likely in the presence of reinforcing factors such as high parental accommodation, anxiety, or inconsistent feeding structure.

Importantly, the boundaries between stages are not defined by the number of foods accepted alone, but by the degree of functional impact and persistence over time (Table 1). A child with a limited diet but preserved growth and minimal family disruption may fall within a less severe phenotype, whereas a child with similar dietary restriction accompanied by significant distress, rigidity, or social limitation may be positioned further along the continuum [10,11].

## 4. Epidemiology and Measurement Challenges

### 4.1. Prevalence Variability

Reported prevalence of picky eating varies widely, reflecting differences in age, population, definitions and assessment tools [1,4]. Prevalence is generally highest between 2 and 6 years of age, coinciding with developmental neophobia and increasing autonomy around eating [1]. Substantial variability even within similar age groups suggests that methodological factors play a major role [4].

Longitudinal studies highlight the distinction between point prevalence and persistence. Although many children are identified as picky eaters at a given time point, only a minority continue to display marked food selectivity over several years [6,8]. This limits the clinical usefulness of cross-sectional prevalence estimates.

### 4.2. Limitations of Current Tools

Most studies rely on parent-reported questionnaires to identify picky eating [1,4]. Wardle et al. developed the Children’s Eating Behaviour Questionnaire [16], while Van Strien et al. proposed the Dutch Eating Behavior Questionnaire [17]. These tools frequently amalgamate food neophobia, low appetite, sensory sensitivity, and oppositional behavior, and their cut-off points are rarely validated against clinical or functional outcomes [4,7].

In addition, most instruments were developed for descriptive research rather than clinical decision-making and offer limited insight into severity, persistence, or functional impact [1,7]. Outcomes such as family stress, mealtime disruption, and social participation are seldom captured, perpetuating the gap between epidemiology and clinical relevance [5].

An additional limitation is the lack of alignment between research-based definitions and clinically meaningful assessments. In practice, clinicians are less concerned with whether a child meets a specific questionnaire threshold and more with whether food selectivity results in nutritional compromise, functional impairment, or family distress. This discrepancy highlights the need for pragmatic assessment frameworks that integrate quantitative indicators of dietary variety with qualitative clinical judgment. Incorporating simple markers—such as number of accepted foods, degree of rigidity, and impact on family functioning—may provide a more clinically relevant approach than reliance on existing research tools alone [1,4,7].

### 4.3. Cultural and Parental Bias

Parental perception plays a central role in identifying picky eating and brings important cultural and contextual influences [1,9]. Norms related to food variety, portion size, and mealtime structure shape what parents perceive as problematic [1,9]. Parental anxiety and feeding style further influence both reporting and child behavior, often in a bidirectional manner [5,9].

As a result, prevalence estimates reflect not only child behavior but also parental perception and cultural context, limiting generalizability and reinforcing the need for function-oriented assessment approaches [1].

Socioeconomic context and family structure also influence how picky eating is expressed and perceived. Picky eating may be reported less frequently in children from lower socioeconomic backgrounds, likely reflecting reduced food choice, more structured meals, and lower parental accommodation rather than true absence of selective behaviors [4,6]. When present in lower-resource settings, however, its nutritional consequences may be more pronounced and less likely to be recognized clinically [18].

Family size appears to have a moderating effect. Children growing up in larger families, particularly with older siblings, tend to show lower prevalence or persistence of picky eating, possibly due to shared meals, peer modeling, and reduced parental pressure around intake [9].

Daycare or kindergarten attendance also modifies the expression of picky eating. Children exposed to group-based feeding environments are less frequently described as picky eaters, and when selectivity is present, it tends to be less persistent [4,6]. Structured routines, repeated exposure to foods, and peer modeling likely contribute to this effect. Importantly, improved intake in daycare settings does not exclude clinically relevant picky eating when difficulties persist at home but rather underscores the strong influence of context [19].

Another important consideration is that prevalence estimates may mask meaningful phenotypic heterogeneity. Children classified as picky eaters differ substantially in dietary patterns, sensory sensitivity, appetite regulation, and behavioral responses to food. Grouping these children under a single label limits the ability to identify clinically relevant subtypes and may contribute to inconsistent findings across studies [19]. Future epidemiological work should therefore move beyond prevalence estimates toward phenotype-based characterization [1,4,7].

## 5. Developmental Trajectories and Prognosis

### 5.1. Transient Versus Persistent Picky Eating

Longitudinal research shows that picky eating follows distinct developmental trajectories [6,8]. For many children, food selectivity peaks during the preschool years and gradually improves with time, repeated exposure, and maturation [6]. This transient pattern is generally associated with normal growth and minimal long-term consequences [6,11].

In contrast, a smaller subgroup exhibits persistent picky eating, with stable selective behaviors extending into later childhood or adolescence [8]. Persistence, rather than early presence, is the key determinant of clinical relevance [8].

Persistent picky eating is characterized by stability over time, resistance to exposure, and greater association with functional impairment. Increasing evidence suggests that this phenotype shares features with broader behavioral traits, including heightened sensory sensitivity and vulnerability to anxiety, and may represent a distinct developmental pathway rather than merely a prolonged form of typical picky eating [8,20,21].

From a clinical perspective, this distinction has important implications. Children with persistent picky eating are less likely to respond to reassurance alone and may require earlier, more structured intervention to prevent further entrenchment of selective patterns. In addition, the co-occurrence of sensory sensitivity or anxiety-related traits may influence both the presentation and response to intervention. This suggests that management strategies should be adapted to the child’s broader behavioral profile rather than focusing solely on dietary intake [22].

This phenotype-oriented perspective reinforces the need to move beyond a dual classification of picky versus non-picky eating and instead consider developmental trajectory, persistence, and functional consequences when guiding clinical decision-making [6,8].

### 5.2. Predictors of Persistence

Predictors of persistence include early severity, extremely limited food repertoires, strong rejection of novelty, and heightened sensory sensitivity [8]. Temperamental traits such as behavioral inhibition and vulnerability to anxiety also play a role [8]. Parental pressure, coercive feeding practices, and feeding-related anxiety are associated with maintenance of picky behaviors through bidirectional pathways [5,9]. Conversely, structured, low-pressure feeding environments may mitigate persistence and support gradual expansion of dietary variety.

Neurodevelopmental conditions and medical comorbidities further increase the likelihood of persistence [10].

### 5.3. Associations with Later Outcomes

Transient picky eating has not been consistently associated with adverse growth or health outcomes [6,11]. In contrast, persistent picky eating is associated with lower dietary variety and increased risk of micronutrient inadequacy [4,7]. Associations with anxiety and internalizing behaviors have been reported, although causality remains uncertain [8]. Family stress and mealtime conflict are among the most consistent adverse outcomes [5,9].

## 6. Mechanisms and Drivers

Picky eating arises from the interplay of biological predisposition, developmental processes, and environmental influences [19]. Genetic studies indicate a substantial heritable component to picky eating and food fussiness, particularly for food neophobia and sensory-based avoidance [20,21,23]. Sensory sensitivity to taste and texture is a common driver of food selectivity, especially in children with neurodevelopmental conditions [8]. Learning mechanisms are central: repeated neutral exposure increases acceptance, whereas pressure-based feeding reinforces avoidance [2,3]. Temperamental traits such as anxiety and behavioral inhibition further amplify avoidance responses [8]. Healthcare professionals should also remain alert to medical contributors—including gastroesophageal reflux, food allergy, eosinophilic gastrointestinal disorders, and constipation—which may trigger food refusal in a minority of cases and warrant targeted evaluation when red flags are present [10].

Importantly, these mechanisms rarely operate in isolation. Picky eating is best understood as the result of dynamic interactions between child characteristics and the feeding environment [24]. For example, a child with heightened sensory sensitivity may be more vulnerable to developing persistent food selectivity in the context of high parental accommodation or pressure, whereas the same child in a structured, low-pressure feeding environment may show gradual improvement. This interactional perspective reinforces the importance of considering both intrinsic and environmental factors when evaluating and managing picky eating [2,3,8].

Taken together, these findings highlight that picky eating is best understood as a dynamic and multifactorial process rather than a single, isolated behavior. Biological predispositions may shape initial responses to food, while environmental influences—including caregiver practices and feeding context—determine whether these tendencies are reinforced or attenuated over time. This interactional model helps explain the marked heterogeneity observed among children with picky eating and underscores why similar behaviors may follow very different developmental trajectories [24].

From a clinical standpoint, this perspective supports a more individualized approach to management, in which both child characteristics and environmental factors are considered, rather than relying on uniform strategies across all cases [2,3,8].

## 7. Clinical Impact

Persistent picky eating is associated with reduced dietary diversity and increased risk of micronutrient inadequacy, even in children with normal growth [4,7]. Associations with underweight or overweight are inconsistent and largely confined to severe cases or those overlapping with feeding disorders [11,14]. This variability reflects the heterogeneity of picky eating and reinforces the importance of distinguishing between transient and persistent patterns when evaluating clinical relevance.

Beyond micronutrient intake, picky eating may influence broader dietary patterns. Children with persistent selectivity often consume fewer fruits, vegetables, and protein-rich foods, while relying more heavily on energy-dense, highly palatable foods [25]. Although growth is typically preserved in milder cases, these patterns may have implications for long-term health trajectories and the development of enduring dietary habits [7,11].

Over time, these patterns may contribute to the consolidation of food preferences that persist into later childhood and adolescence. Early dietary experiences play a critical role in shaping long-term eating behaviors, and limited exposure to a variety of foods during sensitive developmental periods may reduce acceptance of those foods later in life [26]. Although the direct impact on long-term health outcomes remains an area of ongoing research, these trajectories raise concerns about the potential for enduring dietary imbalance.

Importantly, these effects are not uniform across all children with picky eating but are more likely to be observed in those with persistent and more restrictive patterns. This further emphasizes the need to distinguish between transient and persistent phenotypes when considering clinical relevance and the potential need for intervention [19].

The psychosocial impact of picky eating is often more pronounced and consistent than its nutritional consequences. Family stress, mealtime conflict, and parental anxiety are frequently reported and may contribute to a cycle of maladaptive feeding interactions [5,9]. These dynamics can affect family functioning and reduce the quality of shared mealtime experiences [3,9,20].

In addition, picky eating may limit social participation, particularly in settings where food is a central component of interaction, such as school, daycare, or social gatherings [5,20]. Children may avoid eating in unfamiliar environments or experience distress when exposed to non-preferred foods, further reinforcing selective patterns.

Overall, the clinical significance of picky eating depends largely on its persistence, severity, and functional impact. Distinguishing between transient, developmentally typical selectivity and more entrenched patterns associated with nutritional risk or psychosocial burden is essential for appropriate clinical decision-making and for avoiding both unnecessary intervention and under-recognition of clinically relevant cases.

## 8. Clinical Implications and Closing Remarks for Pediatric Practice

Despite the growing volume of research on picky eating, clear and immediately actionable advances for parents and children remain limited. Much of the existing evidence has improved conceptual understanding but has not simplified day-to-day clinical decision-making or reduced parental uncertainty. Families continue to receive mixed messages, ranging from reassurance alone (“it’s just a phase”) to premature concern and intervention.

For pediatricians, picky eating remains a common but challenging presentation. A central task is determining when food selectivity reflects developmentally typical behavior and when it represents a clinically relevant condition requiring further evaluation or intervention. This distinction is critical, as both under-recognition and over-medicalization carry risks.

A structured clinical approach may include: (i) assessment of growth and nutritional adequacy; (ii) identification of red flags suggesting underlying medical or developmental conditions; (iii) evaluation of feeding dynamics and family context; and (iv) determination of persistence and functional impact to guide management and referral decisions [10]. This approach aligns with the proposed continuum model and supports more individualized clinical care.

When children eat a wider variety of foods in daycare or kindergarten than at home, this should not be interpreted as the absence of picky eating, but rather as evidence that structured, low-pressure environments can temporarily attenuate selective behaviors.

Pediatricians must therefore be confident that picky eating is indeed the primary issue and not a manifestation of an underlying organic condition. Careful clinical assessment—including attention to growth patterns, dietary history, red-flag symptoms, developmental status, and family context—remains essential. Conditions such as gastrointestinal disease, food allergy, feeding skill disorders, and chronic discomfort should be thoughtfully excluded before labeling a child as a picky eater.

Importantly, variability in eating behavior across settings can provide useful clinical insight. Children who eat a wider variety of foods in daycare or school than at home should not be considered free of picky eating; rather, this pattern highlights the influence of structured, low-pressure environments in modulating selective behaviors [27].

Contextual factors should also be considered during clinical evaluation. Picky eating may be more difficult to recognize in lower-resource settings, where limited food choices and structured routines may mask selective behaviors, yet nutritional consequences may be more significant. Conversely, in highly accommodating environments or in single-child families, selective eating may be more persistent and reinforced [28].

Management should be guided by severity, persistence, and functional impact rather than by the mere presence of food refusal. Developmentally typical picky eating generally requires reassurance and anticipatory guidance, whereas persistent or functionally impairing patterns may benefit from structured behavioral strategies or referral to multidisciplinary care. Clear communication with families is essential to align expectations and reduce unnecessary anxiety while ensuring that clinically relevant cases are appropriately addressed. Practical strategies include repeated, non-pressured exposure to foods, structured meal routines, and avoidance of coercive feeding practices [10,18].

In cases where food selectivity is severe, persistent, or associated with significant nutritional compromise or psychosocial impact, referral to multidisciplinary care should be considered [29]. This may include collaboration with dietitians, feeding therapists, psychologists, or other specialists, depending on the underlying drivers of the child’s eating behavior. Early referral in appropriate cases may help prevent further progression and reduce the burden on both the child and family.

At the same time, it is important to avoid unnecessary escalation in children with developmentally typical picky eating, where reassurance and anticipatory guidance remain sufficient. Harmonious balance is central to effective clinical management and reinforces the value of a structured, continuum-based approach [3,10].

Ultimately, improving care for children with picky eating will require not only better conceptual frameworks but also practical, accessible strategies that can be implemented across diverse clinical settings [30].

## 9. Future Directions

Future research should move beyond prevalence estimates toward clinically meaningful and implementable frameworks. Progress in the field will depend on developing definitions that incorporate persistence, severity, and functional impact, rather than relying solely on the presence of food refusal. Similarly, outcome measures should extend beyond anthropometric indices to include dietary diversity, family functioning, and social participation.

Advancing the field will also require phenotype-informed approaches to intervention. Recognizing the heterogeneity of picky eating, future studies should move away from uniform management strategies and instead evaluate targeted interventions based on underlying drivers such as sensory sensitivity, anxiety, or behavioral patterns. Longitudinal research linking early phenotypes to treatment response will be essential to support more individualized care pathways.

At the same time, implementation research should become a central priority. A key challenge is not only generating evidence, but ensuring that evidence-based feeding guidance can be effectively integrated into routine pediatric practice, particularly in primary care and in low-resource settings where access to specialized services may be limited.

Finally, greater conceptual alignment between picky eating, pediatric feeding disorder, and avoidant/restrictive food intake disorder is needed to support coherent clinical pathways across the spectrum of selective eating. Without this integration, advances in understanding are likely to continue outpacing improvements in everyday care [3,10]. Ensuring that future developments translate into meaningful benefits for children and families will require practical, adaptable, and context-sensitive approaches to care [19,31].

## Figures and Tables

**Table 1:** Phenotype-Based Continuum of Picky Eating in Children.

Stage	Characteristics	Growth	Functional Impact	Clinical Approach
Typical picky eating	Age-appropriate selectivity	Normal	Minimal	Reassurance
Persistent picky eating	Limited variety, resistance	Usually normal	Moderate (family/mealtime)	Behavioral guidance
Severe/selective eating	Highly restricted intake	May be affected	High	Multidisciplinary
ARFID/PFD	Disorder-level presentation	Compromised	Severe	Specialized care

## Data Availability

No new data were created or analyzed in this study. Data Sharing is not applicable to this article.
